# Differential Effects of UCHL1 Modulation on Alpha-Synuclein in PD-Like Models of Alpha-Synucleinopathy

**DOI:** 10.1371/journal.pone.0034713

**Published:** 2012-04-13

**Authors:** Anna E. Cartier, Kiren Ubhi, Brian Spencer, Ruben A. Vazquez-Roque, Kori Ann Kosberg, Lawrence Fourgeaud, Priya Kanayson, Christina Patrick, Edward Rockenstein, Gentry N. Patrick, Eliezer Masliah

**Affiliations:** 1 Department of Neurosciences, School of Medicine, University of California San Diego, La Jolla, California, United States of America; 2 Department of Pathology, School of Medicine, University of California San Diego, La Jolla, California, United States of America; 3 Departamento de Fisiología, Escuela Nacional de Ciencias Biológicas, Instituto Politecnico Nacional, Mexico City, México; 4 Molecular Neurobiology Laboratory, The Salk Institute of Biological Studies, La Jolla, California, United States of America; 5 Division of Biological Sciences, Section of Neurobiology, University of California San Diego, La Jolla, California, United States of America; Hertie Institute for Clinical Brain Research and German Center for Neurodegenerative Diseases, Germany

## Abstract

Parkinson's disease (PD) is a progressive neurodegenerative disorder caused by genetic and environmental factors. Abnormal accumulation and aggregation of alpha-synuclein (a-syn) within neurons, and mutations in the a-syn and UCH-L1 genes have been shown to play a role in the pathogenesis of PD. In light of recent reports suggesting an interaction between a-synuclein and UCH-L1, we investigated the effects of UCH-L1 inhibition on a-syn distribution and expression levels in primary neurons and hippocampal tissues derived from non transgenic (non tg) and a-syn over expressing tg mice. We show that suppression of UCH-L1 activity increased a-syn levels in control, non tg neurons, and resulted in a concomitant accumulation of presynaptic a-syn in these neurons. In contrast, blocking UCH-L1 activity in a-syn over expressing neurons decreased a-syn levels, and enhanced its synaptic clearance. In vitro studies verified the LDN-induced inhibition of UCH-L1 had minimal effect on LC3 (a marker of autophagy) in control cells, in cells over expressing a-syn UCH-L1 inhibition resulted in increased LC3 activity. These findings suggest a possible differential role of UCH-L1 function under normal and pathological conditions. Furthermore, in the context of a-syn-induced pathology, modulation of UCH-L1 activity could serve as a therapeutic tool to enhance the autophagy pathway and induce clearance of the observed accumulated/aggregated a-syn species in the PD brain.

## Introduction

Parkinson's disease (PD) is a neurodegenerative disorder that affects 1–2% of the population over age 60 [1], [2]. PD is characterized by progressive accumulation of a-synuclein (a-syn) in cortical and subcortical regions leading to neuronal degeneration culminating in motor dysfunctions and dementia [3], [4]. To date several genes including that encoding a-syn have been conclusively linked to PD [5]. However, the link to ubiquitin carboxyl-terminal hydrolase L1 (UCH-L1), remains controversial. UCH-L1 is a multi-functional protein and is highly abundant in the brain [6]. UCH-L1, through its hydrolase activity, is responsible for generating free monomeric ubiquitin from precursor poly-ubiquitin chains [6] and can also associate with mono-ubiquitin to inhibit its degradation and, therefore, stabilize and maintain ubiquitin levels [7]. UCH-L1 also acts as a ligase *in vitro*, and such ligase activity has been demonstrated towards a-syn [8].

The implied involvement of UCH-L1 in PD is based on the discovery of a I93M mutation in the *UCH-L1* gene reported in a German family with autosomal dominant PD [9]. The UCH-L1^I93M^ mutant displays significant decreased hydrolase activity *in vitro*, implying that loss of UCH-L1 function may contribute to a decrease in the availability of free ubiquitin, and an impaired clearance of proteins by the Ubiquitin Proteasome System (UPS). Furthermore, transgenic mice that over express the I93M mutant, show behavioral and pathological deficits characteristic of Parkinsonism [10]. A previous report demonstrated that UCH-L1 is localized to synaptic vesicles, and co-immunoprecipitates with a-syn from rat brains [8]. a-syn is enriched in pre-synaptic terminals, and is potentially involved in synaptic plasticity and neurotransmitter release [11]. Moreover, a-syn is a major component of pathogenic insoluble proteinaceous deposits known as Lewy bodies (LBs) commonly found in PD brains [12]. *In vitro*, UCH-L1 was found to possess a dimerization-dependent ligase activity responsible for K63-linked polyubiquitination of a-syn [8], which may potentially block its degradation and lead to its accumulation and aggregation within neurons. Interestingly, a polymorphism in the UCH-L1 gene (S18Y), was found to be associated with a lower risk of developing PD, has a significantly reduced ligase activity leading to reduced levels of ubiquitinated a-syn [8], [13], [14]. However, more recent studies have failed to find significant association between S18Y polymorphism and reduced PD risk [15], [16].

We have previously shown that inhibition of UCH-L1 activity leads to dysregulation of monomeric ubiquitin homeostasis and dramatic alterations in synapse structure [17]. The aim of our present study was to investigate the effects of UCH-L1 inhibition on a-syn. We show that pharmacological suppression of UCH-L1 activity has differential effects on a-syn distribution and protein expression levels in hippocampal tissues derived from non tg vs. a-syn tg mouse model. Similar differential effects on a-syn distribution was observed in primary hippocampal neuron cultures expressing endogenous a-syn or over expressing human (h)a-syn. Interestingly, these alterations correlate with alterations in the molecular components of the autophagy pathway in a-syn tg mice. These data point to a possible differential role of UCH-L1 function under normal and pathological conditions.

## Materials and Methods

### Reagents

UCH-L1 (LDN-57444 (LDN)) inhibitor was purchased from Calbiochem (San Diego, CA). The HA-tagged Ubiquitin probe (HAUb-VME; vinyl methyl ester functionalized probe) was synthesized as described previously [18], and was provided by Dr. H. Ovaa (Netherlands' Cancer Institute, Netherlands).

### Antibodies

The following antibodies were used in this study: mouse anti-PSD-95 (Calbiochem, San Diego, CA); rabbit anta-synapsin I (Invitrogen, Camarillo, CA); chicken anti-Map2 (Abcam, Cambridge, MA); rabbit anti-UCH-L1 (Biomol, Plymouth Meeting, PA); rabbit anti-Ubiquitin (Dako, Carpinteria, CA); mouse anti-HA (Covance, Emeryville, CA); mouse anti-a-syn (BD Transduction Laboratories, San Jose, CA); rabbit anti-a-syn (Chemicon, Bedford, MA); mouse anti-a-syn (Syn211) (Sigma, Saint Louis, MO); rabbit anti-P62 (MBL, Japan), and rabbit anti-cleaved LC3 (Abgent, San Diego, CA).

### Primary neuronal cultures

Hippocampal neuron cultures were prepared from P1 CD-1 mice or transgenic mice over expressing human a-syn tagged to GFP at its C-terminus (h-a-syn-GFP tg) [19]. Briefly, mouse hippocampi were dissected in HBSS dissecting media containing 4 mM NaHCO3 (7.5%) and 10 mM HEPES buffer. Neurons were then dissociated by enzymatic treatment with 0.25% trypsin in dissecting media for 15 min at 37°C, and subsequent mechanical trituration. For immunostaining, neurons were plated at medium density (45,000 cells/cm^2^) on coverslips (12 mm in diameter) coated with poly-D-lysine. Cultures were maintained in B27 supplemented Neurobasal media (Invitrogen) until 19–21 days *in vitro* (DIV).

### Silencing UCH-L1 using LV-siRNA

The siUCHL1 lentivirus vectors were constructed with the following sequence ACA GGA AGT TAG CCC TAA A (#2) corresponding to nucleotides 385–403 or GCA GCT TTA GCA CTT AGA A (#4) corresponding to nucleotides 909–927 of mouse UCHL1 mRNA. The shRNA sequences were cloned into the pSIH1-copGFP vector (SBI Biosystems) to generate LV-siUCHL1#2 and LV-siUCHL1#4 vectors. A control siRNA vector was generated by cloning the sequence CGT GCG TTG TTA GTA CTA ATC CTA TTT designed against the sequence of luciferase (SBI Biosystems) into the same vector to generate pLV-siLuc. Lentiviruses expressing siUCHL1#2 or #4, a-synuclein (a-syn) or empty vector (LV-Control) were prepared by transient transfection in 293T cells [20].

To determine the effects of down-regulation of UCHL1 on accumulation of a-syn, B103 cells were plated on poly-lysine coated glass coverslips and infected with either LV-Control or LV-a-syn and then co-infected with either LV-siUCHL1#2 or LV-siUCHL1#4 for 72 hours. Cells were then fixed in 4% PFA and processed for immunoflouresence.

### Animals

For the purpose of this study, 6 months old mice over expressing human (h) a-syn under the control of the murine (m) Thy-1 promoter were used [21]. ha-syn accumulation in mThy-1 tg mice occurs at synapses as well as in cell bodies of pyramidal neurons in the cortex and the hippocampus. This model was selected because mice from this line develop widespread intraneuronal a-syn aggregates distributed throughout the neocortex and hippocampus similar to what has been observed in Dementia with Lewy bodies (DLB). All experiments described were approved by the animal subjects committee at the University of California at San Diego (UCSD) and were performed according to NIH recommendations for animal use. UCSD is an Institutional Animal Care and Use Committee accredited institution. The protocol used for this study (protocol ID#S02221) was approved by UCSD Animal Subjects Committee and was followed in all studies according to the Association for Assessment and Accreditation of Laboratory Animal Care International guidelines.

### Drug treatments

For immunofluorescence staining experiments *in vitro*, cultured neurons were treated with 10 µM of UCH-L1 inhibitor (LDN) for 24 hr. This dose was selected based on previous IC50 studies that have shown a specific for LDN to UCH-L1 at this concentration [22]. For protein expression analysis by Western blotting or immunofluorescence staining experiments *in vivo*, wild type and a-syn transgenic mice were injected with LDN (0.5 mg/Kg) intraperitoneally at time zero. Mice were injected again with the same dosage of LDN four hours after the first injection. Mice were then sacrificed four hours after the second injection. This dose of LDN was chosen for *in vivo* injections based on similar doses reported previously [22].

### DUB labeling assay

To investigate levels of UCH-L1 activity we measured the activity of deubiquitinating enzymes (DUB). The DUB activity assay was done as previously described [17], [18]. Briefly, the DUB activity assay was done by incubating 10 µg of hippocampal lysates with the HAUb-VME substrate in labeling buffer (50 mM Tris, pH 7.4, 5 mM MgCl2, 250 mM sucrose, 1 mM DTT, and 1 mM ATP) for 1 h at 37°C. Proteins were then resolved on SDS-PAGE 4–20% gradient gels, and blots were subsequently probed with anti-HA monoclonal antibody. Labeled proteins were identified based on their migration on SDS-PAGE gels, and by comparison to previous published data where the specific bands were analyzed by mass spectroscopy [23].

### Neuronal cell line culture and LDN treatment in vitro

The rat neuroblastoma cell line B103 was used for in vitro experiments [24]. B103 cells were plated at 3.5E4 cells/well on coverslips. After 6 hours cells were infected with LV-control, LV-a-syn and LV-LC3-GFP (MOI = 50) and treated a further 72 hours later with LDN (10 nM, 12 hrs). Cells were then fixed in 4% paraformaldehyde and subsequently analyzed for the expression of a-syn (mouse anti-a-syn (1∶250)) and LC3 (rabbit anti-cleaved LC3 (1∶500)) as described below. Cytotoxicity was assessed using the lactate dehydrogenase (LDH, CytoTox 96 assay, Promega) and MTT (3-(4,5-Dimethylthiazol-2-yl)-2,5-diphenyltetrazolium bromide, Roche) cell viability assays, as per manufacturer's instructions, to measure levels of cell death.

### Immunostaining of neurons in culture

At the end of each experiment, hippocampal neurons plated on coverslips were rinsed briefly in PBS and fixed with 4% paraformaldehyde (PFA) and 4% sucrose in PBS-MC (phosphate buffered saline with 1 mM MgCl2 and 0.1 CaCl2) for 10 min at room temperature. Neurons were then rinsed 3× with PBS-MC and subsequently blocked and permeabilized with blocking buffer containing (2% BSA, 0.2% Triton* X-100 in PBS-MC) for 20 min. After rinsing neurons 3× with PBS-MC, primary antibodies were added in blocking buffer (without 0.2% Triton* X-100) and cultures were incubated overnight at 4°C. The following antibodies and dilutions were used for immuno-fluorescence staining: mouse anti-PSD-95 (1∶1000), rabbit anti-synapsin I (1∶5000), rabbit anti-UCH-L1 (1∶2500), chicken anti-Map2 (1∶5000), and mouse anti-a-syn (1∶2500) and rabbit-a-syn (1∶2500). After three washes with PBS-MC, neurons were incubated in goat anti-rabbit, -mouse or -chicken secondary antibodies conjugated to Alexa 488, Alexa 568, or Alexa 678 (1∶500 each; Molecular Probes) at room temperature for 1 hr. Neurons were washed 3× with PBS-MC and mounted on slides with Aqua Poly/Mount (Polysciences, Warrington, PA).

### Image acquisition and analysis of immunostained neurons in culture

Confocal images were acquired using a Leica (Wetzlar, Germany) DMI6000 inverted microscope outfitted with a Yokogawa (Tokyo, Japan) Spinning disk confocal head, a Orca ER High Resolution B&W Cooled CCD camera (6.45 µm/pixel at 1X) (Hamamatsu, Sewickley, PA), Plan Apochromat 40×/1.25 na and a Melles Griot (Carlsbad, CA) Argon/Krypton 100 mW air-cooled laser for 488/568/647 nm excitations. Exposure times were held constant during acquisition of all images for each experiment. Pyramidal-like cells were chosen in a random fashion. Confocal z-stacks were taken at 0.4–0.5 µm depth intervals in all experiments. For image analysis, the NIH ImageJ software was utilized. Max Z-projected images were thresholded equally 1.5–2 times above background. Dendrites from individual neurons were then straightened and used for analysis. Fluorescence intensity associated with pre- and postsynaptic protein puncta was measured to determine the size and number of puncta (normalized to dendritic length) in control and LDN-treated neurons. Statistical significance was determined by unpaired two-tailed Student's t test. All imaging and analysis were performed in a blinded fashion.

### Tissue processing, immunohistochemistry and image analysis

At the end of each experiment, mouse brains were removed and divided sagittaly. One hemibrain from each brain was snap-frozen and stored at −70°C for protein and RNA analysis, while the other hemibrain was fixed in PBS/4% PFA (pH 7.4) at 4°C. 40 µm thick free-floating Vibratome sections were immunolabeled with the mouse anti-a-syn (1∶2500) and/or rabbit anti-UCH-L1 antibodies. After overnight incubation with the primary antibodies, sections were incubated with the Thyramide Signal Amplification-Direct (Red) System (NEN Life Sciences) and/or FITC-conjugated secondary antibody (Vector laboratories). Sections were then transferred to SuperFrost slides (Fisher Scientific, Tustin, CA), and mounted under glass coverslips with anti-fading media (Vector Laboratories). All sections were processed under the same conditions. Sections were imaged with a Zeiss objective on an Axiovert 35 microscope (Zeiss, Germany) with an attached MRC1024 laser scanning confocal microscope (LSCM) system (BioRad, Hercules, CA). Fluorescence intensity associated with a-syn immunoreactive structures, was measured using NIH ImageJ software. A total of six mice per group and three sections per mouse were analyzed. Four fields in the hippocampus, per section, were examined. Statistical significance was determined by unpaired two-tailed Student's t test. All imaging and analysis were performed in a blinded fashion.

### Stereological analysis

Neurons in the CA1 region of the hippocampus were visualized with Nissl staining. Neuron density measurements were performed using an image-based analysis system. Seven sections per condition were analyzed. The region of interest in each section was selected with a 4× objective on an Olympus BX51 microscope (Olympus Denmark A/S, Denmark). Each region was then divided into randomly selected squares by the Stereo Investigator software (MBF bioscience). An average of 20 squares per selected area in each section was used to count the total number of nucleoli-containing neurons using a 100× oil objective. The total number of neurons was calculated according to the optical fractionator [25]. All imaging and analysis were performed in a blinded fashion.

### Western Blot analysis

Mouse hippocampi were homogenized in RIPA buffer in teflon-glass homogenizers. Hippocampal homogenates were centrifuged at 18,000 g and supernatants were removed and protein concentration was determined with the BCA™ Protein Assay Kit (Pierce, Rockford, IL) using bovine serum albumin as a standard. Protein samples were resolved by SDS-PAGE and electrophoretically transferred to nitrocellulose membranes. Membranes were then blocked for 1 hr in TBST blocking buffer (TBS, 0.1% Tween 20, and 5% milk) at room temperature and then incubated with primary antibodies in blocking buffer overnight at 4°C. The antibodies used were at the following dilutions: rabbit anti-ubiquitin (1∶1000), rabbit anti-P62 (1∶1000), rabbit anti-cleaved LC3 (1∶1000), mouse anti-a-syn (Syn211, 1∶2500) and mouse anti-a-syn (1∶2500). Blots were then washed 3× in TBST washing buffer (TBS, 0.1% Tween 20) and incubated with goat anti-rabbit or -mouse IgG conjugated to horseradish peroxidase (1∶5000). Protein bands were visualized by Chemiluminescence plus reagent (PerkinElmer) and were digitized and quantified using NIH ImageJ software. For statistical analysis unpaired Student t-test was performed between any two conditions.

### Real-time analysis of RNA expression

Hippocampi from non tg and a-syn tg control and LDN-treated mouse brains were isolated. Total RNA from each hippocampus was then extracted with the RNeasy Lipid Tissue kit (Qiagen). cDNA from extracted RNA was generated with the qScript cDNA synthesis kit according to the manufacturor's protocol. cDNA was then quantified using the 2× SYBR Green SuperMix for iQ (Quanta Biosciences) with specific primers for murine a-syn (5′-TGG CAG AAG CAG CAG GAA AG, 3′-AGC CAC TGT TGC CAC ACC AT) and human a-syn (5′-CAG GTC TGA GGC CTC CCT TTT, 3-GCT GCC TCA ACA CCT CAA CC). For statistical analysis one-way ANOVA and post hoc Bonferroni was performed between all conditions.

## Results

### Characterization of a-syn and UCH-L1 distribution in primary neurons

**Figure 1:**
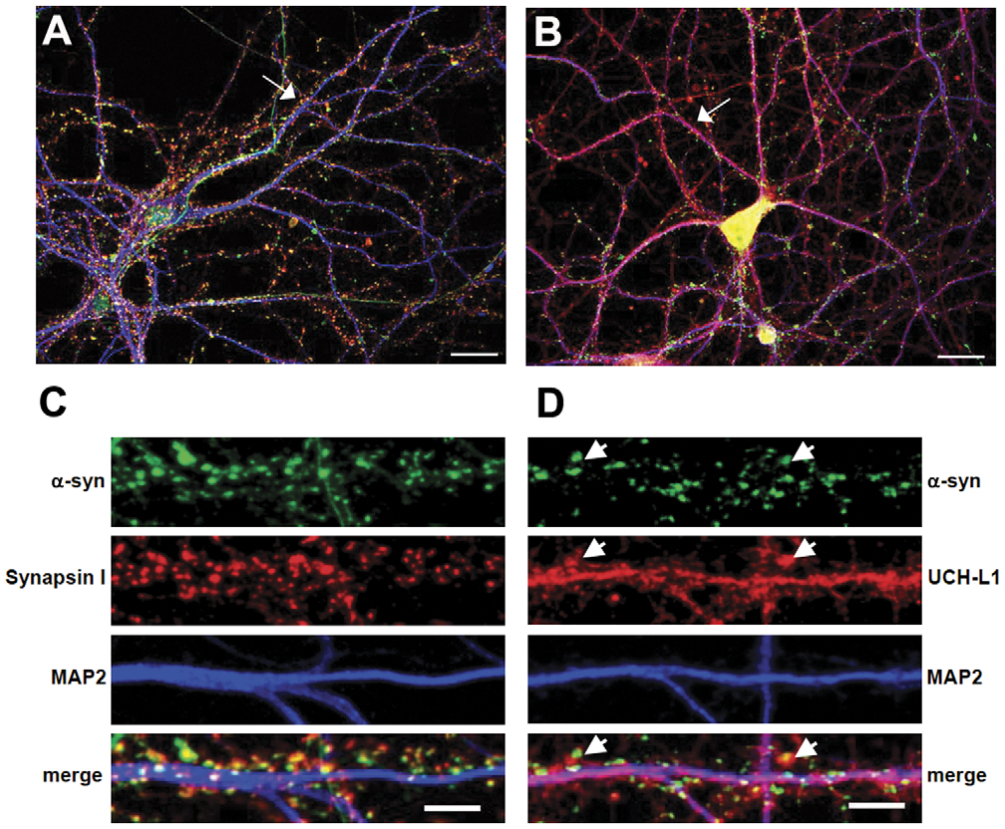
Subcellular localization of a-syn and UCH-L1 in mature cultured hippocampal neurons. Representative images of hippocampal neurons immunolabeled with anti-a-syn, anta-synapsin I and anti-MAP2 antibodies (A, C) or anti-a-syn, anti-UCH-L1 and anti-MAP2 antibodies are shown (B, D). The straightened dendrites in (C) and (D) correspond to the regions indicated by arrows in the whole cell images (A, B). Arrowheads in (D) indicate selected regions where UCH-L1 colocalizes with a-syn. Representative max z-projected confocal images (cell and straightened dendrites) are depicted. Whole cell (A, B) scale bar = 20 µm; dendrite (C, D) scale bar = 5 µm.

In order to determine the subcellular localization of a-syn we immunostained dissociated cultured hippocampal neurons (DIV 20) for endogenous a-syn, the presynaptic marker Synapsin I (Figure 1A) and UCH-L1 (Figure 1B). The MAP2 (dendritic protein) staining was used as a marker for dendritic integrity. expression was detected in both soma and axons of hippocampal neurons. A-syn accumulated at the presynaptic terminals as evidenced by co-localization with Synapsin I, a well-known presynaptic protein (Figure 1A, C). A-syn also partially co-localized with UCH-L1, which is expressed both in the pre- and postsynaptic sites, soma and dendrites of hippocampal neurons (Figure 1B, D) [8], [17]. The observed distribution of a-syn is in agreement with previous reports [26], [27].

### Inhibition of UCH-L1 activity affects a-syn protein clusters in primary neurons

**Figure 2:**
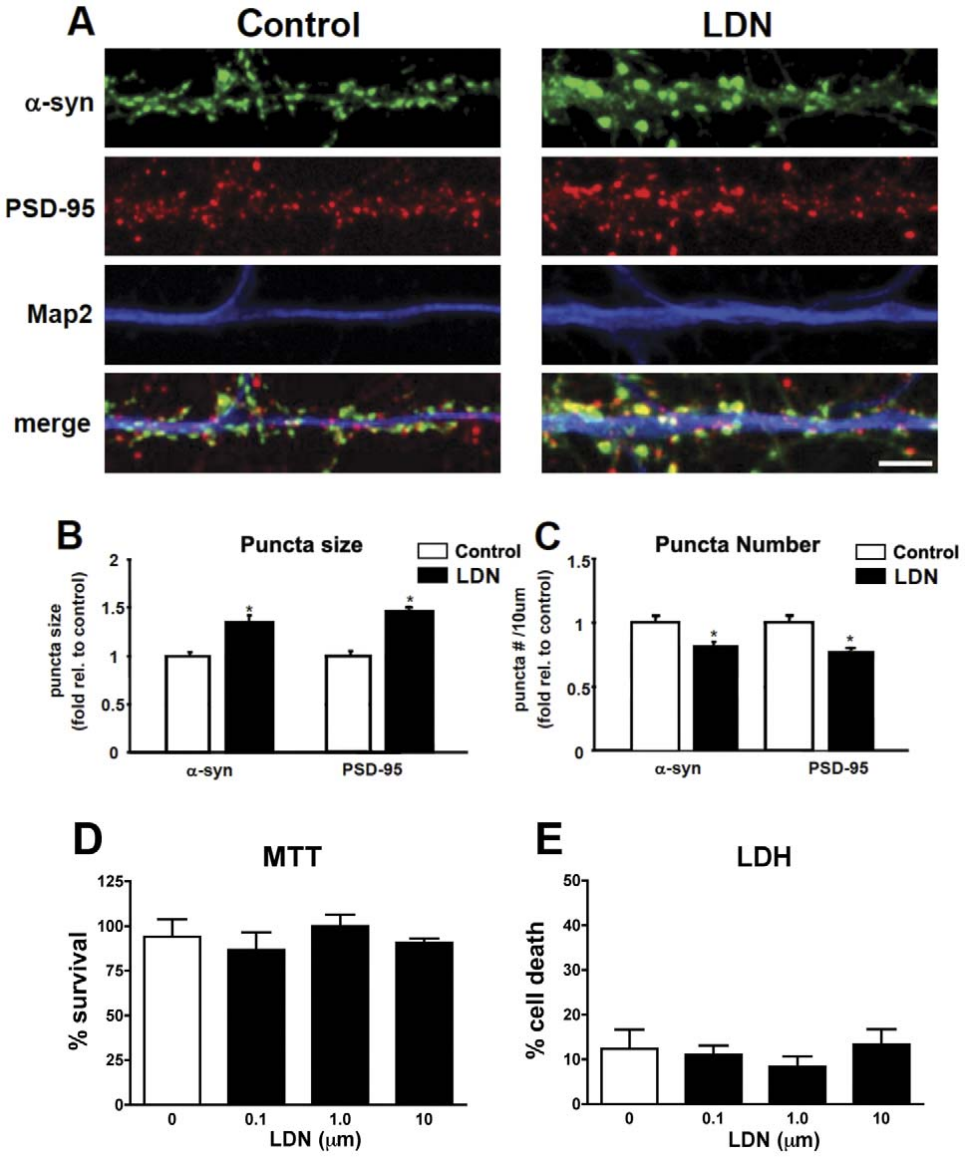
Inhibition of UCH-L1 activity alters a-syn puncta size and number. Cultured neurons were treated with LDN for 24 hours. At the end of LDN treatments, neurons were fixed, permeabilized, and immunolabeled with anti-a-synuclein, anti-PSD-95 and anti-MAP2 antibodies (A). Representative straightened dendrites from control and LDN-treated neurons are depicted. Scale bar = 5 µM. a-synuclein protein puncta size (B) and number (C) were analyzed in control and LDN-treated neurons. The mean puncta size and number in LDN-treated neurons were normalized to those of control neurons from 3 independent experiments. The number of puncta was calculated per 10 µm dendrite length. Measurements for immunostainings were made on greater than 60 dendrites for control and LDN-treated neurons. (D, E) Effects on cell survival as evidenced by the MTT and LDN assays, respectively in cells treated with LDN. Mean values ± SEM are shown. *P<0.05.

Our previous work determined that UCH-L1 activity is required for maintaining synapse structure [17]. Since an interaction between UCH-L1 and a-syn has been reported at synaptic vesicles *in vitro* [8], we assessed whether alterations in UCH-L1 activity had any effect on a-syn distribution. For these experiments we used a previously described UCH-L1 inhibitor, LDN-57444 (LDN), which is known to inhibit UCHL1 activity with an IC50 of 0.88 µM [22], [28]. For these experiments we used LDN at 10 nM, utilizing the Dub labeling assay we found that consistent with previous studies [17] LDN (n = 3, 0.45±0.04) reduced UCH-L1 activity by 48% compared to vehicle control (n = 3, 0.22±0.03) We examined the immunocytochemical distribution of a-syn in control and LDN-treated neurons (Figure 2A). We found that exposure of mature hippocampal neurons to LDN leads to a significant increase in the size of a-syn puncta in LDN-treated neurons as compared to those in control neurons (Figure 2A, B). On average, there was a 35% increase in the size of a-syn puncta (Figure 2B, control, 1.0±0.05; LDN-treated, 1.35±0.07). We also examined whether changes in a-syn puncta size was accompanied by an alteration in the density of these puncta. We found that the density of a-syn puncta was decreased by 19% (Figure 2A, C, control, 1.0±0.04; LDN-treated, 0.81±0.04). We also assessed whether inhibition of UCH-L1 activity had an effect on the size and density of PSD-95 puncta, and found that while the size of PSD-95 puncta increased by 46%, its density was reduced by 24% (Figure 2A–C, PSD-95 puncta size, control, 1.0±0.05; LDN-treated, 1.46±0.05; PSD-95 puncta #, control, 1.0±0.05; LDN-treated, 0.76±0.04). This observation is in agreement with our previous study where we assessed the size and density of PSD-95 puncta in rat hippocampal cultures [17]. Assessment of levels of UCH-L1 immunostaining showed no difference between cells treated with vehicle or LDN (data not shown). There were no observable differences in MAP2 staining between control and LDN-treated neurons (Figure 2A, B), indicating that inhibition of UCH-L1 activity had no effect on the overall integrity of dendrites. Consistent with this finding, MTT and LDH cell viability assays conformed that LDN was not toxic to the cells at the dose chosen for this study (Figure 2D, E).

### Altered UCH-L1 activity affects a-syn distribution in non tg and a-syn tg mice

**Figure 3:**
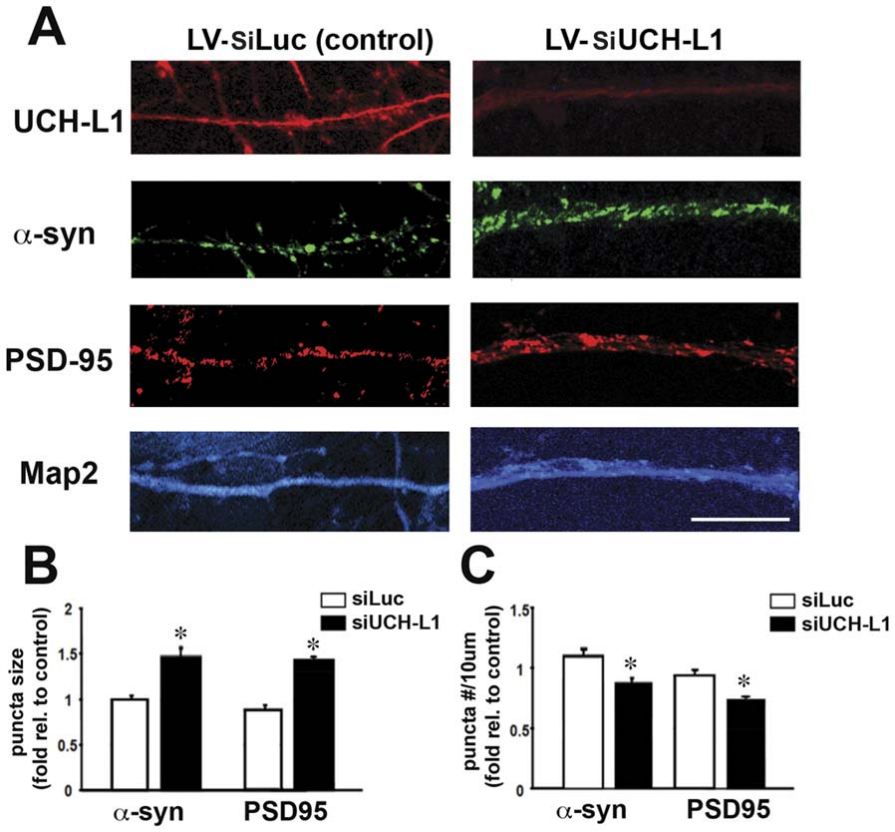
Effects of UCH-L1 signaling on a-syn and PSD-95. To assess the effects of UCH-L1 activity on PSD-95 and a-syn, primary neuronal cultures were infected with a lenti-viral vector expressing a si-control (LV-siLuc) or si-UCH-L1 (LV-siUCH-L1) and then analyzed by immunocytochemistry and confocal analysis (A). a-syn and PSD95 protein puncta size (B) and number (C) were analyzed in neurons expressing a LV-siLuc or LV-siUCH-L1. The mean puncta size and number were normalized to those of control neurons from 3 independent experiments. The number of puncta was calculated per 10 µm dendrite length. *P<0.05.

Next, to confirm whether down regulation of UCH-L1 activity affects PSD-95 and a-syn by an independent method, primary neuronal cultures were infected with a lenti-viral vector expressing a si-control (LV-siLuc) or si-UCH-L1 (LV-siUCH-L1) and then analyzed by immunocytochemistry and confocal analysis. As expected, the LV-siUCH-L1 led to a robust reduction in the expression of UCH-L1 (Figure 3A). Consistent with the studies with LDN (Figure 2), LV-siUCH-L1 increased the size of a-syn and PSD-95 punctae (Figure 3A, B) and reduced the number of punta in the dendrites (Figure 3A, C)

Since our data demonstrate that blocking UCH-L1 activity affects a-syn distribution *in vitro*, we set out to determine whether inhibiting UCH-L1 activity plays a role in modulating a-syn distribution *in vivo*. To test this possibility, we assessed the effects of UCH-L1 inhibition under normal and pathological conditions.

**Figure 4:**
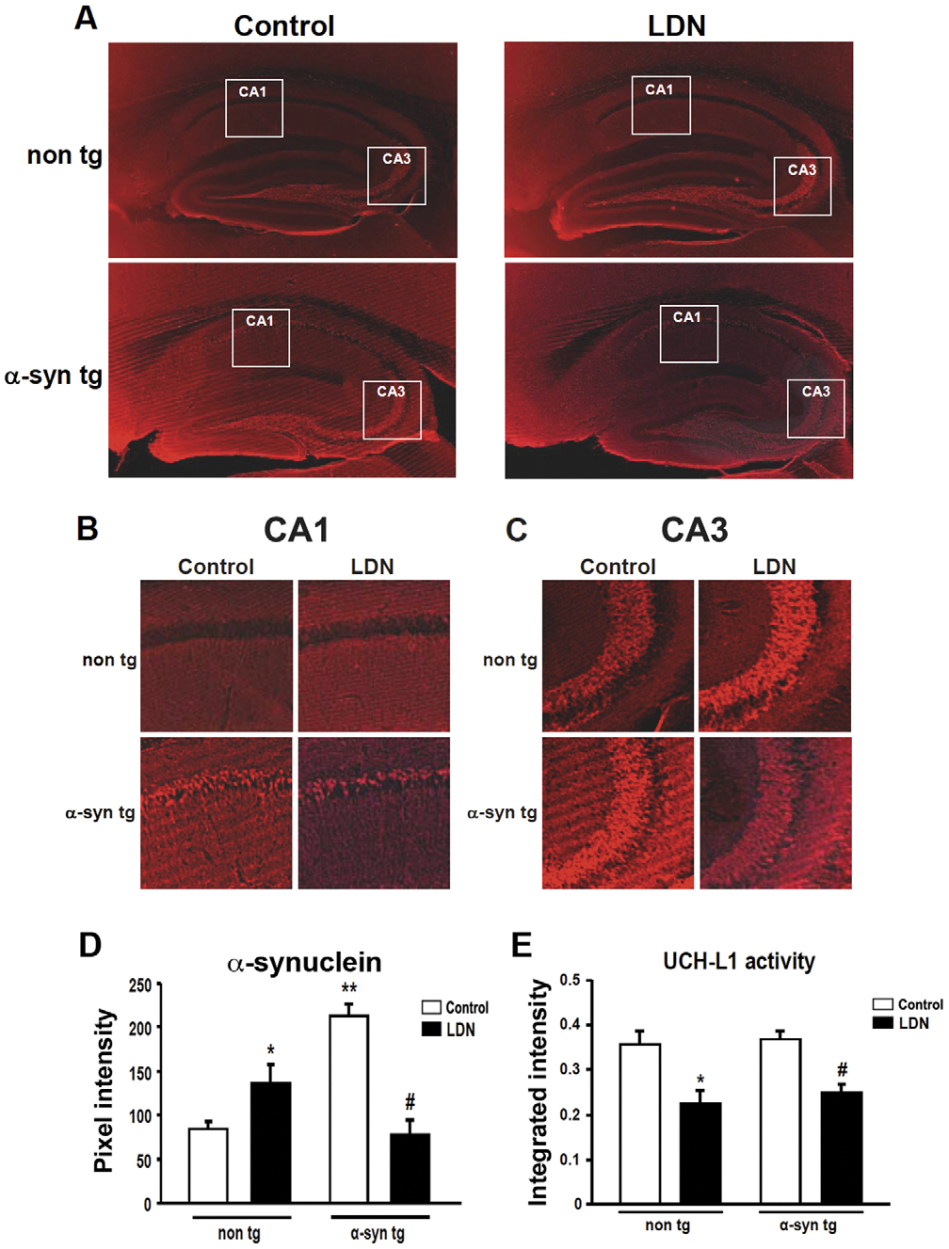
Immunohistochemical analysis of a-syn expression in the hippocampus of control and LDN-treated non tg and a-syn tg mice. Representative hippocampal vibratome sections from control and LDN-treated non tg and a-syn tg mice that were immunolabeled with the anti-a-syn antibody, that recognizes both human and mouse forms of this protein (A). Magnified insets corresponding to boxed areas in the CA1 (B) and CA3 (C) regions from (A) are shown. Mean fluorescence intensity in hippocampal sections from LDN-treated non tg and a-syn tg mice (D). * Indicates a significant difference between non tg mice that were treated with or without LDN P<0.05. ** Indicates significant difference between untreated non tg and a-syn tg mice P<0.05. # Indicates significant difference between a-syn tg mice that were treated with or without LDN P<0.05. Mean values ± SEM are shown. N = 6 mice per group, 3 sections per mouse. UCH-L1 activity in hippocampal homogenates from non tg and a-syn tg mice treated with or without LDN (E). * Indicates a significant difference between non tg mice that were treated with or without LDN P<0.05. # Indicates a significant difference between a-syn tg mice that were treated with or without LDN P<0.01. N = 6 mice per group. Mean values ± SEM are shown. AU = arbitrary unit.

We first examined the immunohistochemical distribution and expression levels of a-syn in hippocampal tissue sections from control and LDN-treated mice. Immunolabeling of hippocampal tissues with a-syn in non tg mice treated with LDN, showed a significant increase in a-syn expression compared to those in control, untreated mice (Figure 4A–D, non tg, control, 1.0±0.1AU; LDN-treated, 1.64±0.3AU). In contrast, a-syn levels were significantly reduced in hippocampal tissues of a-syn tg mice treated with LDN. (Figure 4A–D, a-syn tg, control, 1.0±0.07; LDN-treated, 0.37±0.08). As previously reported, in a-syn tg mice, a-syn immunoreactivity and accumulation was associated with presynaptic terminals, in the neuropil, and in pyramidal cell bodies in the hippocampus [21]. Interestingly, a-syn accumulation or reduction in the CA1 (Figure 4A, B) and CA3 (Figure 4A, C) regions of LDN-treated mice occurred predominantly in the neuropil.

We then determined UCH-L1 activity levels in hippocampal lysates obtained from non tg and a-syn tg mice that received injections of either vehicle (DMSO) or LDN. We found that in the hippocampi of LDN-treated non tg mice, UCH-L1 activity levels were reduced by 37% (Figure 4E, non tg hippocampus, control, 1.0±0.09; LDN-treated, 0.63±0.08). UCH-L1 activity levels were similarly reduced in the hippocampi of a-syn tg mice treated with LDN by 33% (Figure 4E, a-syn tg hippocampus, control, 1.0±0.05; LDN-treated, 0.67±0.05). We did not detect any changes in hippocampal UCH-L1 activity levels between non tg and a-syn tg brains treated with vehicle alone (Figure 4E).

**Figure 5:**
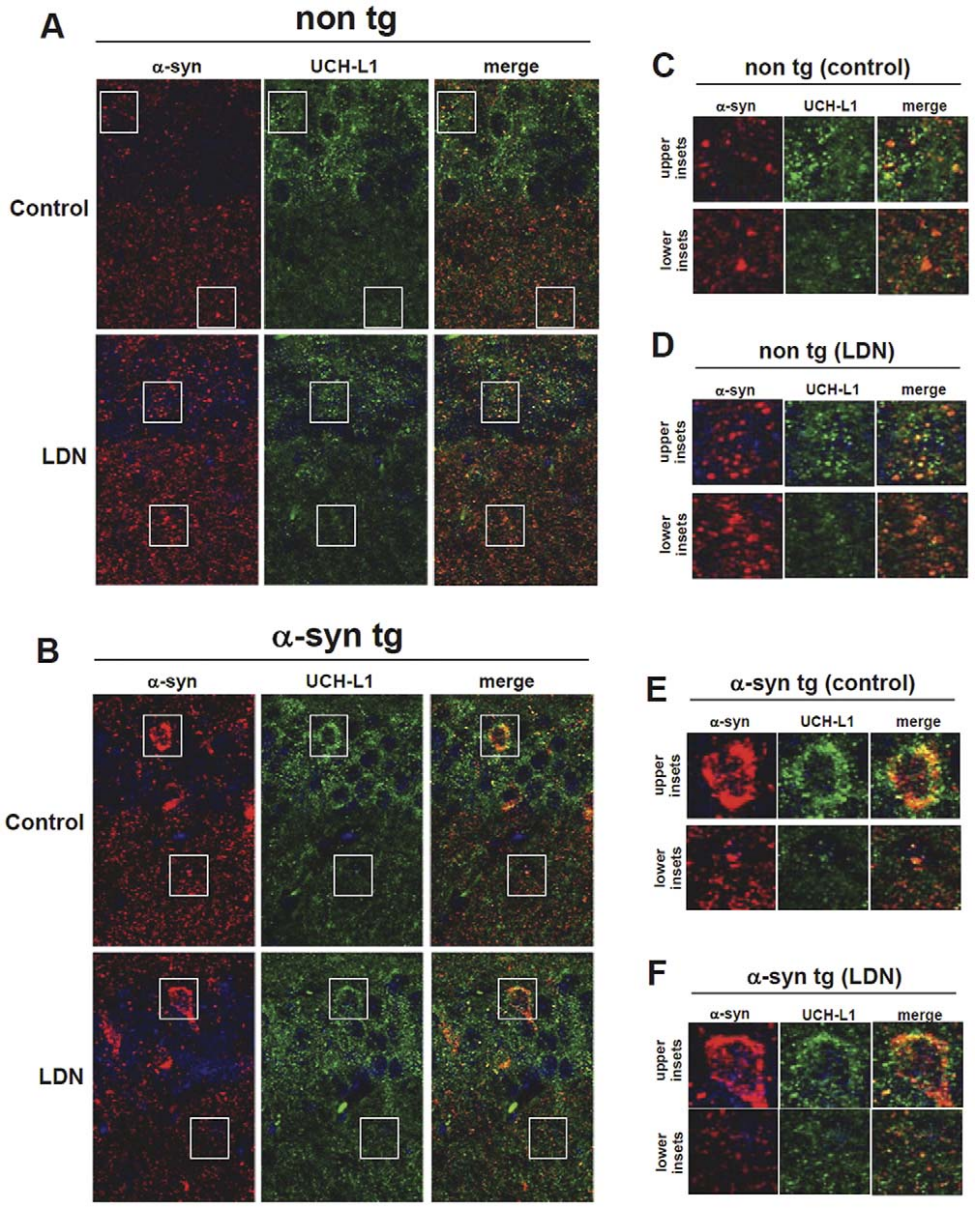
Subcellular localization of a-syn and UCH-L1 in non tg and a-syn tg hippocampal sections. Representative hippocampal vibratome sections from control and LDN-treated non tg (A) and a-syn tg (B) mice that were double-immunolabeled with antibodies against UCH-L1 and a-syn. The antibody against a-synuclein recognizes both human and mouse forms of this protein. Samples were further stained with DAPI to visualize nuclei (blue). Magnified inset correspond to boxed areas from non tg control (C), non tg LDN-treated (D), a-syn tg control (E), and a-syn tg LDN-treated (F) are shown. The upper insets correspond to the upper boxed regions and the lower insets correspond to the lower boxed regions in each representative image.

In order to investigate the relationship between a-syn and UCH-L1 in vivo, detailed confocal imaging was performed on double-labeled sections. High magnification images of double-immunolabeled hippocampal sections with antibodies against a-syn and UCH-L1 demonstrate that in both non tg (Figure 5A, C, D) and a-syn tg (Figure 5B, E, F) hippocampal sections, UCH-L1 is expressed in the soma and is distributed in a micropunctate fashion in the neuropil along a-syn puncta. Interestingly, inhibition of UCH-L1 activity in non tg mice (Figure 5 A, D), led to a more widespread pattern in UCH-L1 staining suggesting redistribution of UCH-L1 in the presence of LDN treatments.

**Figure 6:**
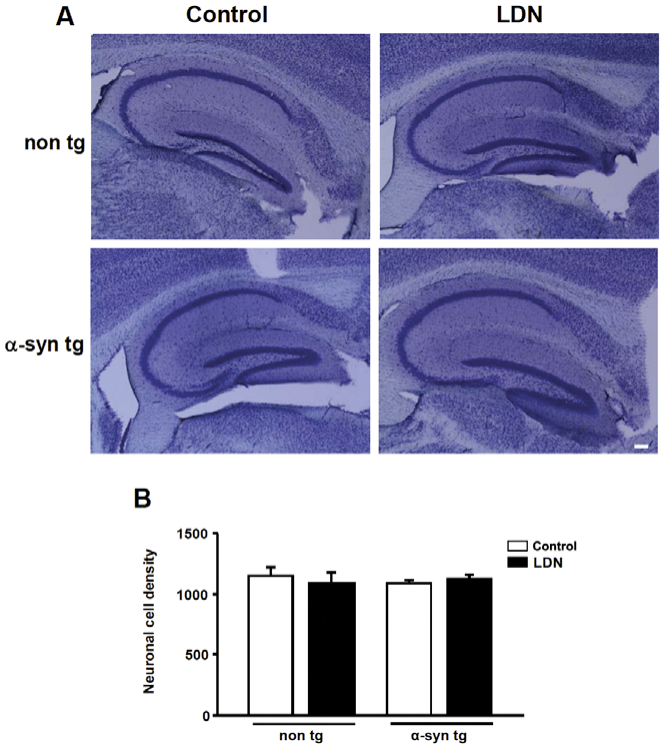
Inhibition of UCH-L1 activity does not alter neuron density in the hippocampus of control and LDN-treated non tg and a-syn tg mice. Neuron density in the CA1 region of the hippocampus was determined by stereological analysis. Representative hippocampal vibratome sections from control and LDN-treated non tg and a-syn tg mice stained with cresyl violet (Nissl) (A). Average neuron density in control or LDN-treated non tg and a-syn tg mice is shown (B). N = 7 mice per group. Mean values ± SEM are shown.

To assess whether the observed alterations in a-syn levels in LDN-treated mice was not due to changes in neuronal cell density, we performed stereological analysis of the hippocampus using Nissl staining (Figure 6A). We did not detect any alterations in neuron density in the CA1 region of LDN-treated mice (Figure 6B, non tg, control, 1.0±0.06; LDN-treated, 0.94±0.08; a-syn tg, control, 1.0±0.03; LDN-treated, 1.04±0.03). Similarly, there were no changes in neuron density in the CA1 region of non tg vs. a-syn tg mice (Figure 6B, non tg, 1.0±0.06; a-syn tg, 0.94±0.03).

### Altered UCH-L1 activity affects a-syn protein levels in non tg and a-syn tg mice

**Figure 7:**
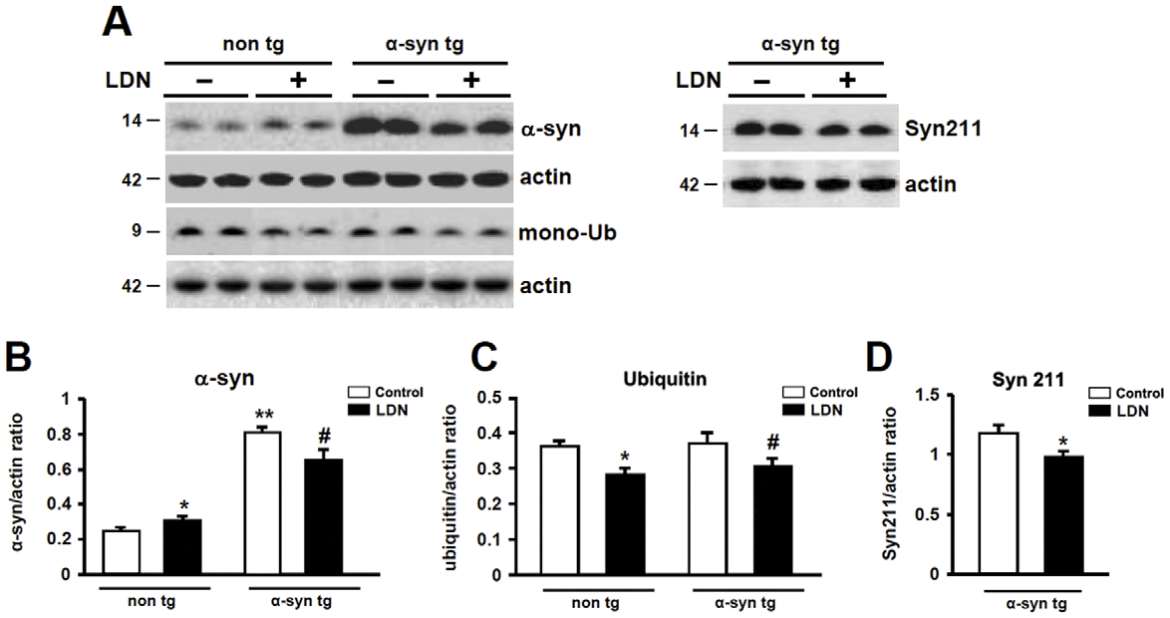
Comparison of a-syn and mono-ubiquitin protein expression levels in hippocampal lysates of control and LDN-treated non tg and a-syn tg mice. Hippocampal lysates from control and LDN-treated mice were analyzed by Western blot (A). Analysis of a-synuclein (murine (B) and human (D)) and ubiquitin (C) levels in control or LDN-treated non tg and a-syn tg mice. * Indicates a significant difference between non tg mice that were treated with or without LDN, P<0.05 and P<0.01for a-syn and ubiquitin expression levels, correspondingly. ** Indicates a significant difference between untreated non tg and a-syn tg mice, P<0.001. # Indicates a significant difference between a-syn tg mice that were treated with or without LDN, P<0.05. (d) Analysis of human a-synuclein levels in control and LDN-treated a-syn tg mice. * Indicates a significant difference between control and LDN-treated a-syn tg hippocampal homogenates, P<0.01. N = 6 mice per group. Mean values ± SEM are shown.

The immunohistochemical data demonstrate that inhibition of UCH-L1 activity has differential effects on a-syn distribution in the hippocampi of non tg vs. a-syn tg animals. To complement the immunohistochemical data, we compared a-syn protein expression levels in hippocampal homogenates obtained from non tg and a-syn tg mice that received injections of either vehicle (DMSO) or LDN. We first assessed whether suppression of UCH-L1 activity had an effect on mono-ubiquitin levels in the hippocampi of LDN-treated mice. We have previously shown that UCH-L1 dependent mono-ubiquitination is a good marker of activity [17]. We found that mono-ubiquitin levels were reduced by 22% in hippocampal lysates of LDN-treated non tg mice (Figure 7A, C, non tg, control, 1.0±0.05; LDN-treated, 0.78±0.05). Similarly, mono-ubiquitin levels were reduced by 18% in hippocampal lysates of LDN-treated a-syn tg mice (Figure 7A, C, a-syn tg, control, 1.0±0.08; LDN-treated, 0.82±0.07). These results support the notion that LDN treatment was active and effective in the mice. There were no detectable differences in mono-ubiquitin levels between non tg and a-syn tg hippocampal lysates (Figure 7A, C). We then analyzed a-syn expression levels with antibodies that recognize both human and murine a-syn (Figure 7A, B) or only human a-syn (Syn211) (Figure 7A, D); the latter would only detect a-syn expression in a-syn tg mice. We found that blocking UCH-L1 activity had differential effects on a-syn expression in non tg vs. a-syn tg mice. In non tg mice, suppression of UCH-L1 activity led to an increase in a-syn protein levels by 25% compared to control untreated mice as measured with the antibody against both human and murine forms of a-syn (Figure 7A, B, non tg, control, 1.0±0.03; LDN-treated, 1.25±0.05). However, blocking UCH-L1 activity in a-syn tg mice reduced a-syn protein levels by 19% (Figure 7A, B, D, a-syn tg, control, 1.0±0.04; LDN-treated, 0.81±0.07). Similar decreased levels in ha-syn protein expression were observed in LDN-treated a-syn tg mice using the Syn211 antibody (Figure 7 A, D a-syn tg, control, 1.0±0.06; LDN-treated, 0.82±0.04). These data suggest that UCH-L1 may function differentially under pathogenic vs. non-pathogenic conditions.

### Inhibition of UCH-L1 activity affects a-syn protein clusters in a-syn over expressing primary neurons

**Figure 8:**
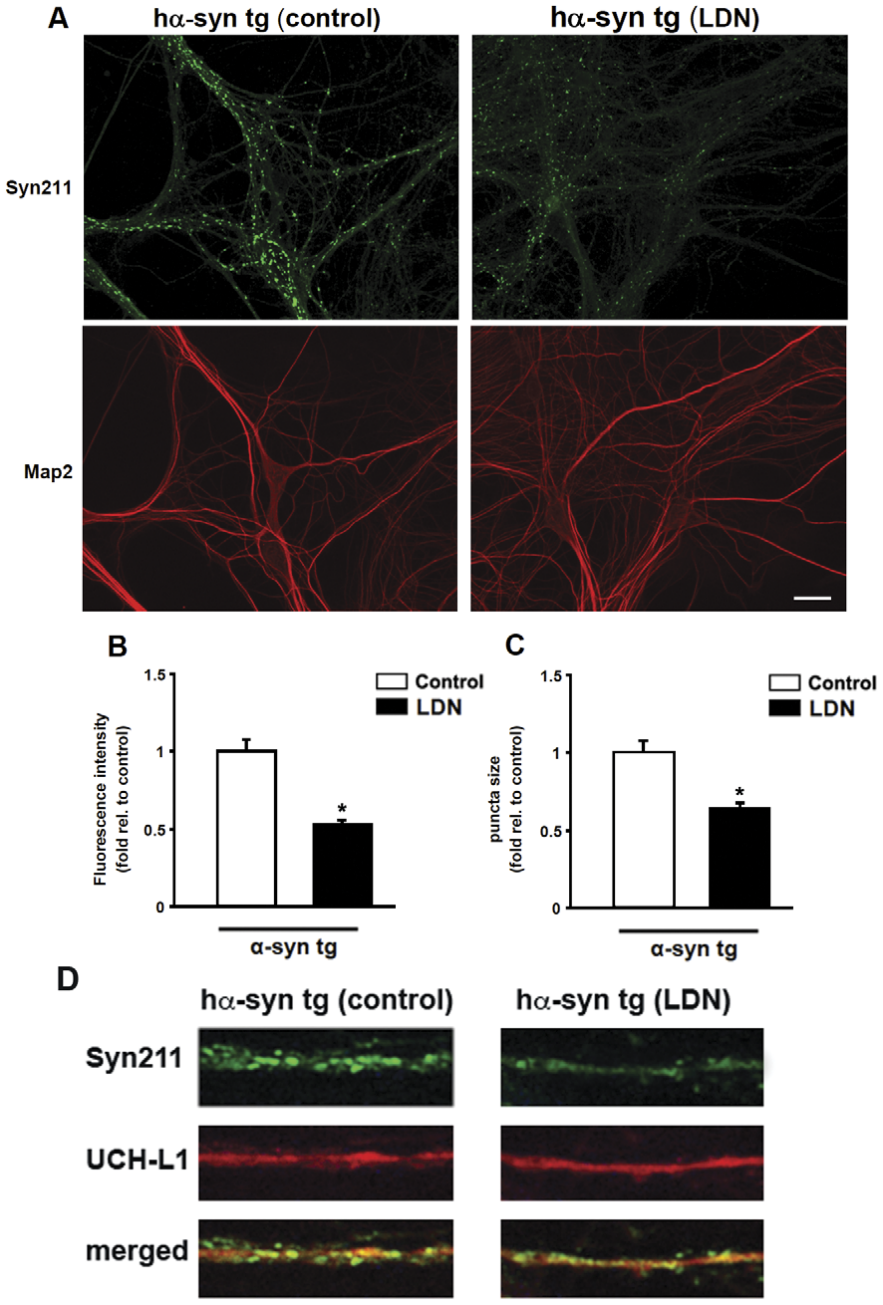
Inhibition of UCH-L1 activity alters a-syn puncta size and intensity in hippocampal primary neurons from h-a-syn-GFP tg mice. Cultured h-a-syn-GFP over expressing hippocampal neurons were treated with LDN for 24 hours. At the end of LDN treatments, neurons were fixed, permeabilized, and immunolabeled with anti-MAP2 and anti-a-syn (Syn211) antibody that only recognizes human a-syn. Representative images from control and LDN-treated neurons (A), scale bar = 20 µM. The mean fluorescence intensity (B) and puncta size (C) in LDN-treated neurons were normalized to those of control neurons. Measurements for immunostainings were made on 10 different fields from 3 independent experiments in both control and LDN-treated neurons. Mean values ± SEM are shown. *P<0.01.

Biochemical and immunohistochemical data from our *in vivo* studies show that the levels of a-syn are modulated differentially in non tg and a-syn tg mice with suppressed UCH-L1 activity. In order to validate our *in vivo* results from a-syn tg mice, we used an independent *in vitro* model to test the effects of UCH-L1 suppression in a-syn over expressing neurons treated with LDN. We used cultured primary hippocampal neurons from h-a-syn-GFP tg mice for this purpose as transgenic mice are readily detected and distinguished from non tg mice using a GFP flashlight. In this mouse model, h-a-syn expression is driven by platelet-derived growth factor (PDGF) promoter which restricts h-a-syn expression largely to the limbic and cortical areas [19], [21]. These mice show striking deficits in spatial memory as determined by the Morris water maze testing [29]. *In vitro*, average h-a-syn expression levels in mature synaptic boutons was ∼2.5 times higher than that of wild type littermates, levels that reasonably approximate those observed in a-syn related human diseases [29]. In addition, brain homogenates from these mice only show a single band corresponding to the expected molecular weight of the h-a-syn-GFP fusion protein, unlike previous reports where proteolytic cleavage products were observed under transient transfections with C-terminally tagged h-a-syn [30]. Thus, in this model, C-terminal tagging of a-syn with GFP does not appear to alter the biophysical properties of the protein. As expected, a-syn was robustly expressed and enriched at synapses (Figure 8A). Consistent with our *in vivo* results, exposure of h-a-syn-GFP neurons to LDN led to significant reduction in the intensity and size of h-a-syn-immunoreactive puncta (Figure 8A–C). On average, we found that the fluorescence intensity and size of a-syn puncta were reduced by 48% and 36%, respectively, in LDN-treated neurons (Figure 8B, C, fluorescence intensity, control, 1.0±0.08; LDN-treated, 0.52±0.03; puncta size, control, 1.0±0.08; LDN-treated, 0.64±0.04). Next we investigated whether LDN treatment modifies UCH-L1 distribution in the ha-syn tg mice. Double-labeling studies demonstrated that UCH-L1 levels of immunostaining were similar between vehicle- and LDN-treated tg mice (Figure 8D). Collectively, these data show that loss of UCH-L1 activity in h-a-syn-GFP over expressing neurons affects the levels and distribution of a-syn.

### Assessment of hippocampal a-syn mRNA levels in non tg and a-syn tg mouse brain

**Figure 9:**
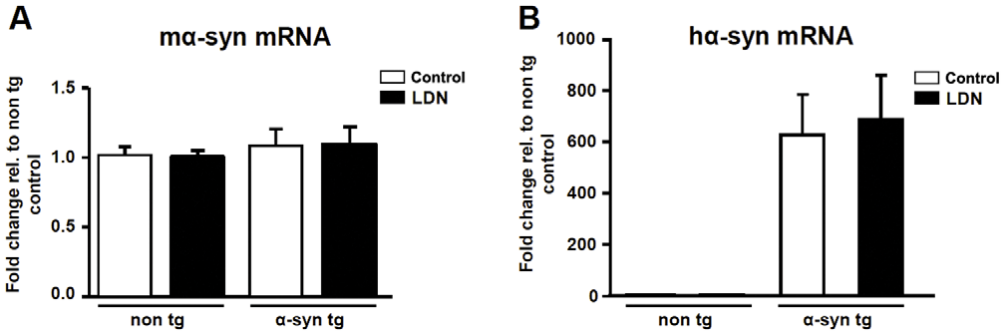
Inhibition of UCH-L1 activity does not affect hippocampal mRNA expression levels of a-syn. Total RNA was extracted from hippocampal tissues of non tg and a-syn tg mice treated with or without LDN, and analyzed by real-time PCR for expression of murine a-syn (ma-syn) (A) and human a-syn (ha-syn) (B). a-syn mRNA expression levels for all groups were normalized to those in control non tg mice. N = 6 mice per group. * Indicates a significant difference between ha-syn mRNA levels in untreated non tg and a-syn tg mice, P<0.0001. Mean values ± SEM are shown.

The observed alteration in a-syn protein expression levels could be attributed to either a change in the stability of this protein or its mRNA expression levels. In order to test these possibilities, we examined endogenous m-a-syn and h-a-syn mRNA levels in hippocampal tissues from non tg and a-syn tg mouse brains. No differences were detected in m-a-syn or h-a-syn mRNA levels, between control or LDN-treated non tg and a-syn tg hippocampi (Figure 9A, B). Our mRNA analysis results suggest that the observed alteration in a-syn protein expression levels are not due to alterations in mRNA expression levels.

### Effects of UCH-L1 inhibition on molecular components of the autophagy pathway

**Figure 10:**
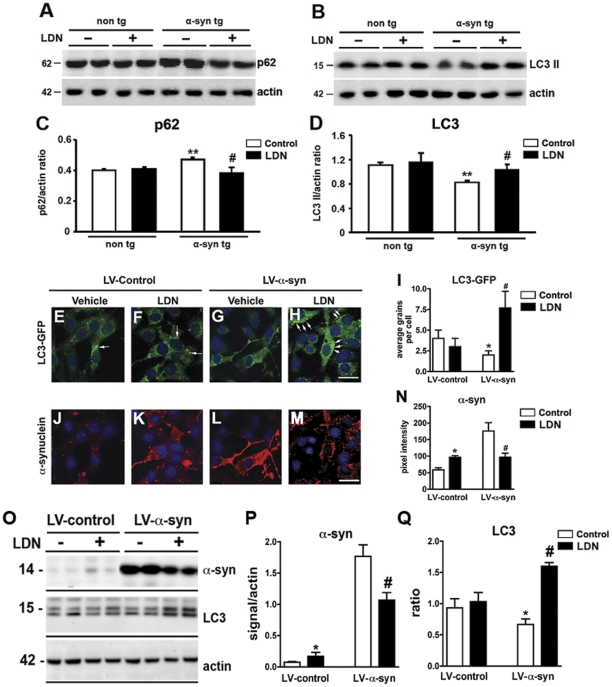
Effects of altered UCH-L1 activity on markers of autophagy. Immunoblot analysis for p62 (A) and LC3I and II (B) of hippocampal homogenates from control or LDN-treated non tg and a-syn tg mice. Quantitative analysis of p62 levels in hippocampal homogenates (C). Quantitative analysis of the ratio of LC3II/I levels in hippocampal homogenates (D). ** Indicates a significant difference between untreated non tg and a-syn tg mice, P<0.01. # Indicates a significant difference between control and LDN-treated a-syn tg mice, P<0.05. B103 rat neuronal cells infected with LV-LC3-GFP and LV–control or LV-a-syn were treated with vehicle or LDN and levels of LC3-GFP signal (E–H) or a-syn (J–M were assessed by immunohistochemistry. Quantitative analysis of LC3-GFP signal in cells (I). Quantitative analysis of a-syn immunoreactivity in neuronal cells (N). Representative immunoblot for a-syn, LC3 and actin in rat neuronal cells infected with LV–control or LV-a-syn and treated with vehicle or LDN (O). Analysis of levels of a-syn and LC3 II/I ratio (P–Q). * Indicates a significant difference between vehicle and LDN-treated LV-control infected cells P<0.01. # Indicates a significant difference between vehicle and LDN-treated LV-a-syn infected cells, P<0.05.

To further analyze the differential effects of UCH-L1 inhibition in LDN-treated mice, and delineate possible mechanism(s) by which UCH-L1 leads to modulation of a-syn protein expression levels, we set out to examine components of the autophagy pathway. Recent studies have demonstrated that UCH-L1 interacts with the components of the chaperone-mediated autophagy (CMA), a pathway also known to be involved in degradation of a-syn [31], [32]. We examined protein expression levels of cleaved LC3 (LC3 II) and p62. LC3 is a marker for autophagy as full length LC3 (LC3 I) is cleaved to form LC3 II when autophagy is induced [33], [34]. p62 is an ubiquitin and LC3-binding protein, and has been shown to target ubiquitinated proteins for degradation by the proteasome and autophagy [35], [36], [37], [38], [39]. Interestingly, the levels of p62 itself can also be regulated by autophagy. We did not detect significant differences in either p62 (Figure 10A, C) or LC3 II (Figure 10B, D) levels in non tg mice treated with or without LDN. However, we observed an accumulation in p62 protein in the hippocampal homogenates of control, untreated, a-syn tg mice compared to non tg controls (Figure 10A, C, non tg, control 1.0±0.02; a-syn tg, control, 1.18±0.02). Suppression of UCH-L1 activity in a-syn tg mice, however, decreased p62 levels by 19%, and to levels observed in non tg mice (Figure 10A, C, a-syn tg, control 1.0±0.0; LDN-treated, 0.81±0.07).

Assessment of cleaved LC3 levels in hippocampal homogenates showed that compared to control non tg mice, the levels of LC3 II decreased by 26% in a-syn tg mice (Figure 10B, D, non tg, control 1.0±0.04; a-syn tg, control, 0.74±0.03). Inhibition of UCH-L1 activity in a-syn tg mice increased in LC3 II levels by 45% (Figure 10B, D, a-syn tg, control 1.1±0.05; LDN-treated, 1.55±0.1). These data suggest that inhibition of UCH-L1 activity induces activation of the autophagy pathway in a-syn tg mice but not in non tg mice.

In order to verify the effects of UCH-L1 inhibition on autophagocytic markers in an independent system, a neuronal cell culture model of a-syn over expression was employed. B103 cells, a rat neuronal cell line, were co-infected with LV-LC3-GFP and either a LV–control construct or LV-a-syn and subsequently treated with LDN (Figure 10 E–N). Levels of LC3-GFP signal in the LV-Control infected cells were not significantly affected upon LDN treatment (Figure 10E, F, I). However, levels of a-syn were increased after LDN treatment in control cells (Figure 10K, N). In contrast, the effects of UCH-L1 inhibition on levels of a-syn *in vitro* were consistent with those observed *in vivo* and demonstrate that LDN treatment resulted in a decrease in a-syn (Figure 10M, N) and an increase in LC3-GFP signal in the cells infected with LV-a-syn, this LC3-GFP signal was localized to discrete granular structures in the cell. (Figure 10 G, H, I). These results were confirmed by immunoblot (Figure 10O–Q). Collectively these data suggest that inhibition of UCH-L1 activity induces activation of the autophagy pathway in a-syn tg mice and a-syn over expressing cells but not in non tg mice or controls cells suggestive of a differential effect of UCH-L1 modulation under pathogenic vs. non-pathogenic conditions

## Discussion

Cognitive decline and dementia are among the most common nonmotor changes in neurodegenerative disorders such as PD and DLB and are often associated with intraneuronal a-syn deposits which lead to synaptic dysfunction and degeneration [40], [41]. Elevations in a-syn protein levels contribute to the pathogenesis observed in PD, as gene duplications/triplications of the a-syn gene have been shown to cause familial PD or parkinsonism [42], [43]. In the present study, we set out to study the relationship between UCH-L1 and a-syn as these proteins are implicated in PD and have been previously shown to interact [8]. We examined hippocampal tissues to assess potential UCH-L1-dependent alterations that may occur in this brain region due its inactivation. In doing so, we investigated mechanisms that could potentially lead to neuronal dysfunction and loss and ultimately cognitive decline and dementia. Here we show that inhibition of UCH-L1 activity in non tg mice leads to an increase in a-syn protein expression levels. Similarly, inhibition of UCH-L1 activity in primary hippocampal neurons expressing endogenous levels of a-syn led to an increase in the size and number of presynaptic a-syn clusters. In contrast, blocking UCH-L1 activity in a-syn transgenic mice or neurons that over express a-syn resulted in a decrease in a-syn levels that correlated with its enhanced clearance from synapses. Furthermore, the observed alterations in a-syn levels were not due to changes in its mRNA expression levels or neuron cell density but correlated with dysregulation of the autophagy pathway in a-syn transgenic mice. Our data point to differential effects of UCH-L1 inhibition in the context of normal and pathologic conditions, in that blocking UCH-L1 activity leads to opposing effects on a-syn distribution and protein levels in cells that express endogenous a-syn levels vs. those that over express the protein.

The precise nature of, and mechanisms underlying, how UCH-L1 might modulate a-syn are not clear. One possibility might involve a direct interaction. a-syn is a primarily pra-synaptic protein whilst UCH-L1 is localized both in the pre and post-synaptic regions [8], [17]. Interestingly, in a previous study [17] we have shown that the effects of LDN were preferentially on post-synaptic proteins such as PSD-95. However, as we show here, presynaptic proteins such as a-syn might also be affected.

A recent study examined synuclein levels in ubiquitin carboxyl-terminal hydrolase L1-deficient gad mouse and reported β- and a-synuclein immunoreactive spheroids in the gracile nucleus in these mice [44]. In contrast, alpha-synuclein immunoreactivity was barely detectable in spheroids. However, it is worth noting that they did not investigate a-syn levels in the synapses which where we found re-distribution of a-syn rather than in axonal swellings. In addition, other factors that might explain the differences between our results and theirs are compensatory mechanisms in the context of a developmental UCH-L1 KO compared to a functional transient effect of a pharmacological inhibitor such as LDN.

In our previous study, we demonstrated that pharmacological suppression of UCH-L1 activity led to reduction in mono-ubiquitin levels, which in turn suppressed ubiquitin-dependent protein degradation by the proteasome [17]. Consequently, UCH-L1-dependent perturbation of proteasome activity affected the stability of important synaptic scaffold proteins, PSD-95 and Shank, which are known targets for ubiquitination and degradation by the UPS [17], [45], [46], [47]. Here, we show that reduction in UCH-L1 activity, and subsequent decreased mono-ubiquitin levels, under non-pathogenic conditions, results in accumulation of a-syn, which is degraded by the UPS [48], [49]. These observations are in line with our previous study and demonstrate that alterations in ubiquitin homeostasis could affect ubiquitin-dependent degradation of certain proteins, including a-syn, by the UPS.

In contrast to our observations in non-pathogenic conditions, inhibition of UCH-L1 activity under pathogenic conditions (h-a-syn over expression) reduces a-syn protein levels, and this could potentially be beneficial for neuronal survival. Many lines of evidence have suggested that dysfunction in the autophagy pathway is common in many neurodegenerative disorders including in PD and DLB [50], [51], [52], [53]. Furthermore, a-syn aggregates have been shown to interfere with the autophagy mechanisms and ultimately lead to neurodegeneration *in vivo* and *in vitro* [54], [55], [56]. Interestingly, excess a-syn and its aggregates are cleared by autophagy [49], [57], [58]. This observation is in line with a recent study by Spencer et al., where it was shown that LV-mediated over expression of beclin 1, a major component in initial steps of the autophagy pathway, reduced abnormal accumulation of a-syn and associated neuronal deficits in a transgenic mouse model of DLB [55]. The observed hippocampal accumulation of p62, whose levels are known to increase in response to inhibition in autophagy, with a concurrent decrease in cleaved LC3 II levels in our a-syn transgenic model point to a dysfunction in the autophagy pathway in these mice [59], [60]. Furthermore, Kabuta et al. have shown that UCH-L1 interacts with members of the chaperon-mediated autophagy (CMA), and that the familial PD-associated UCH-L1^I93M^ interacts abnormally with these members, leads to inhibition of CMA and as a result an increase in a-syn levels in cultured cells [61]. Taken together, under pathological conditions (h-a-syn over expression), blocking UCH-L1 activity may enhance autophagy, which in turn will serve to degrade and reduce a-syn levels. The mechanism(s) by which inhibition of UCH-L1 by LDN affects its interaction with the members of autophagy pathway are unknown. Interestingly, our double-immunolabeling for UCH-L1 and a-syn, only in non tg mice treated with LDN, showed a redistribution in UCH-L1 staining pattern. It is unclear how this redistribution may affect the interaction of UCH-L1 with members of the autophagy pathway, and whether these alteration lead to the opposing effects on a-syn observed in non tg vs. a-syn tg mice. Our data suggest that loss of UCH-L1 function in the context of h-a-syn-induced pathologies may have neuroprotective effect in the hippocampus.

A recent study by Liu et al. demonstrated that a population of UCH-L1 is membrane-bound (UCH-L1^M^), and C-terminal farnesylation of UCH-L1 promotes its association with cellular membranes [62]. Over expression of wild type UCH-L1 led to an increase in the levels of UCH-L1^M^ expressed in cells and correlates with accumulation of a-syn and neurotoxicity [62]. Interestingly, a mutation in the farnesylation sequence (CKAA→SKAA) of UCH-L1, resulting in the C220S mutant, eliminates the membrane-associated species of UCH-L1, and has no effect on a-synuclein levels. Pharmacological inhibition of UCH-L1 farnesylation, on the other hand, was shown to reduce a-syn levels possibly by promoting its degradation through lysosomal pathway, and increased cell viability [62]. In addition, approximately 30% of UCH-L1 was found to be membrane-associated in cortical tissues of diseased and normal human brains (e.g. PD and AD). However, an association between UCH-L1^M^/UCH-L1^S^ (soluble UCH-L1) ratio and disease, in this case, in cortical tissues from PD patients was not detected [62]. These data clearly demonstrate a link between UCH-L1 farnesylation, membrane association and a-syn expression levels. However, the effects of modulation of UCH-L1 activity (e.g. by LDN) on these conditions are unknown. It is worth noting that the C220S mutation does not affect hydrolytic activity of UCH-L1 [62]. Interestingly, Kabuta et al. did not report an increase in a-syn expression levels due to wild type UCH-L1 over expression using the same cell culture model system [61]. Also, we have used a model system in which UCH-L1 and a-syn are abundantly expressed in the hippocampus and cultured neurons contrary to these other systems where neither proteins were endogenously expressed. Our use of a system where proteins of interest are endogenously expressed presents a more suitable system and reflective of what may occur *in vivo*.

In conclusion, we show that suppression of UCH-L1 activity has differential effects on a-syn in neurons that express normal or excessive levels of a-syn. Under normal physiological conditions, perturbation of UCH-L1 activity has a dramatic effect on distribution and protein levels of presynaptic a-syn, which in turn could have detrimental effects on normal neuronal function. Under pathological conditions, however, loss of UCH-L1 function proves to be beneficial as it not only enhances a-syn degradation but also relieves the a-syn-mediated block of the autophagy pathway. Further studies are needed to evaluate the potential value of blocking UCH-L1 as a therapeutic target in a-synucleinopathies.
