# Expression of concern: Differential effects of UCHL1 modulation on alpha-synuclein in PD-like models of alpha-synucleinopathy

**DOI:** 10.1371/journal.pone.0319504

**Published:** 2025-02-21

**Authors:** The PLOS One Editors

Following the publication of this article [1], concerns were raised regarding results presented in Figs 7 and 10. Specifically, there appear to be vertical discontinuities in multiple panels presented in Figs 7A, 10A, and 10B.

The corresponding author stated that the blot images presented in the article were for illustrative purposes only, and commented that the analysis was carried out separately. They provided the individual-level data underlying the graphs presented in Fig 7B–D (S1 File), but in the absence of the original blot image data the reliability of the quantified results cannot be verified and the above western blot concerns remain unresolved.

The corresponding author stated that the remaining underlying data for this article are no longer available.

The *PLOS One* Editors issue this Expression of Concern to notify readers that the western blot results presented in this article should be interpreted with caution, and to relay the available underlying data.

## Supporting information

S1 File Individual-level data underlying the quantified western blot results presented in Fig 7B-D. (XLSX)

S2 File Individual-level data underlying UCHL1 LV-syn cells results presented in Figs 10I, and 10N. (XLSX)
